# Morphology and Release Kinetics of Protein-Loaded Porous Poly(L-Lactic Acid) Spheres Prepared by Freeze-Drying Technique

**DOI:** 10.5402/2011/490567

**Published:** 2011-08-15

**Authors:** Takashi Sasaki, Kazuki Tanaka, Daisuke Morino, Kensuke Sakurai

**Affiliations:** Department of Materials Science and Engineering, University of Fukui, 3-9-1 Bunkyo, Fukui 910 8507, Japan

## Abstract

Freeze-drying a biodegradable polymer, poly(L-lactic acid) (PLLA), from 1,4-dioxane solutions provided very porous spherical particles of ca. 3 mm in radius with specific surface area of 8–13 m^2^ g^−1^. The surface of the particle was found to be less porous compared with its interior. To apply the freeze-dried PLLA (FDPLLA) to drug delivery system, its morphology and drug releasing kinetics were investigated, bovine serum albumin (BSA) being used as a model drug compound. Immersion of FDPLLA into a BSA aqueous solution gave BSA-loaded FDPLLA, where mass fraction of the adsorbed BSA reached up to 79%. Time-dependent release profile of BSA in water suggested a two-step mechanism: (1) very rapid release of BSA deposited on and near the particle surface, which results in an initial burst, and (2) leaching of BSA from the interior of the particle by the diffusion process. It was suggested that the latter process is largely governed by the surface porosity. The porosity of both the interior and surface was found to decrease remarkably as the concentration of the original PLLA/1,4-dioxane solution increases, *C*_0_. Thus, *C*_0_ is a key parameter that controls the loading and releasing of BSA.

## 1. Introduction

In recent decades, controlled drug release from capsules made from biodegradable and biocompatible polymers has been extensively studied. Polymer microspheres with dispersed medication are often used for this purpose [1]. In general, the drug release from microspheres are considered to occur both by degradation of the polymer matrix and by simple diffusion of the drug molecules. It has been revealed that various factors affect the feature of drug release, such as the size and structure of the microsphere, porosity, chemistry, degradability, and molar mass of the polymer material, and pH value of the medium [1]. Furthermore, pH-sensitive release technique has been developed [2–4].

Microspheres for the drug release have been prepared by polymerization method or by solvent removing method [1]. Vinyl polymer microcapsules can be prepared by emulsion, suspension, or dispersion polymerization [1, 5]. Furthermore, controlled/living radical polymerization, which has been developed recently, can provide microspheres with well-controlled morphology [6]. On the other hand, for biodegradable polymers such as poly(L-lactic acid) (PLLA), poly(glycolic acid) (PGA), and various naturally occurring polymers, microspheres are usually prepared by solvent evaporation or spray-drying method [7–17].

It needs to be noted that porous biodegradable polymer particles can also be prepared by freeze-drying technique [18], and by solution crystallization method [19]. We have prepared porous PLLA particles by the freeze-drying method from 1,4-dioxane solutions and have investigated their structure and thermal properties precisely [18, 20]. It has been demonstrated that the interior of the freeze-dried PLLA (FDPLLA) is very porous consisting of thin membranes forming a fine, randomly arrayed tissue. In addition, crystallizability of FDPLLA is remarkably high due to the mobile surface layer of the fine membranes, and thus, the range of crystallinity that can be achieved is expanded from that of the bulk PLLA. The FDPLLA seems to be suitable for a carrier in controlled drug delivery system because of its high void content and biocompatibility. The porosity of the FDPLLA is expected to be controlled by freeze-drying conditions. Additionally, it seems that the shape and size of the freeze-dried material can be controlled by the method of freezing. For example, if the solution is frozen as droplets, sphere-shaped material is yielded, and if it is frozen in a space between two flat substrates with a spacer, a plate-like one is yielded.

In this paper, we investigate the possibility of applying the porous FDPLLA to controlled drug release system. We prepare protein-loaded FDPLLA particles with radius of ca. 3 mm and examine their morphology. We here employ a protein, bovine serum albumin (BSA) as a model drug compound. BSA can be loaded into the FDPLLA particle by simply immersing them into a BSA aqueous solution and subsequent drying. The release kinetics of BSA from the BSA-loaded FDPLLA in water is investigated, and the results are discussed in conjunction with the morphology.

## 2. Experimental Methods

### 2.1. Preparation and Characterization of FDPLLA

PLLA with a molar mass of 210 kDa containing 98% L units was supplied from Mitsui Chemicals Co. 1,4-Dioxane was purchased from Nacalai Tesque, which was distilled before use. PLLA/1,4-dioxane solutions of which the PLLA concentration of *C*_0_ = 2.0, 5.0, and 8.0 wt% were prepared. The solution was directly poured into liquid N_2_ (77 K) drop by drop so as to the solution was rapidly frozen. The frozen droplets were dried under vacuum for 168 h to remove the frozen solvent by sublimation, which yielded FDPLLA spherical particles with high porosity. The diameter of the particle was approximately 3 mm, being almost independent of *C*_0_. More detailed procedure of freeze-drying is described elsewhere [18, 20]. Gravimetric measurement after heating at 190°C revealed that the residual solvent contained in the FDPLLA was estimated to be less than 0.1 wt%. We confirmed by differential scanning calorimetry (DSC) and wide-angle X-ray scattering (WAXS) that the as-prepared FDPLLA is completely amorphous as we reported elsewhere [20]. Additionally, we also prepared crystallized FDPLLA by annealing as-prepared FDPLLA at 115°C for 15 min. The crystallinity of the crystallized samples was evaluated from the net heat of endotherm detected during a heating scan of DSC at 10 K min^−1^. Specific surface area of the obtained FDPLLA, *σ*, was evaluated by the Brunauer-Emmett-Teller (BET) adsorption isotherm of krypton. Volume fraction of void in the particle, *r*_*v*_, was also evaluated by measuring the mass and apparent size of the particle. Literature value 1.248 g cm^−3^ for the density of amorphous PLLA was used in this evaluation [21]. Scanning electron microscopy (SEM) was performed to investigate the morphology of FDPLLA particles by using a Hitachi S-2600H electron microscope.

### 2.2. Loading and Releasing of BSA

**Figure 1:**
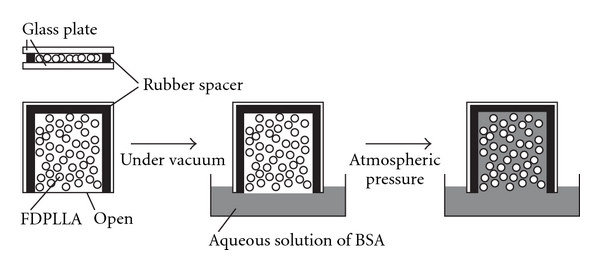
Immersion of FDPLLA particles in BSA aqueous solution by using a cell made of glass plates and a rubber spacer.

BSA (66,776 Da, lyophilized powder, >96%) was purchased from Sigma-Aldrich. FDPLLA was immersed in an aqueous solution of BSA (20 wt%) in the following procedure. FDPLLA particles were placed in a sandwich cell that was made of two glass plates and a rubber spacer and were degassed under vacuum together with a BSA solution in a vacuum chamber (see Figure 1). Then, the open side of the cell was immersed in the BSA solution, followed by releasing the air into the chamber to atmospheric pressure. This method allows the solution to permeate thoroughly into the interior of the FDPLLA particle. On the contrary, when FDPLLA particle was simply added to the BSA solution, the solution hardly permeated into FDPLLA because of very high hydrophobicity of PLLA. After 10 min, the FDPLLA immersed in the solution was then separated and dried at room temperature in atmosphere for 24 h, followed by drying under vacuum for 24 h. The mass of BSA loaded in FDPLLA was evaluated by gravimetric measurement. 

In the release experiments, the ratio of BSA-loaded FDPLLA/water was determined so as to the expected final absorbance of BSA at 279 nm is in the rage 0.5–0.6. In a typical experiment, 50 mg of BSA-loaded FDPLLA with *C*_0_ = 2.0 wt% was immersed in 40 mL of distilled water. The mixture was subject to constant stirring, the temperature being controlled at 37.0 ± 0.1°C. The amount of released BSA was measured by monitoring ultraviolet (UV) absorption at 279 nm, where a peak characteristic of BSA is located with a molar absorption coefficient of 43,824 M^−1^ cm^−1^. The UV measurements were done by using a UV spectrometer Hitachi U-3900 H. From the results, time-dependent profile of the BSA concentration of the aqueous phase was evaluated.

## 3. Results and Discussion

### 3.1. Morphology

**Figure 2:**
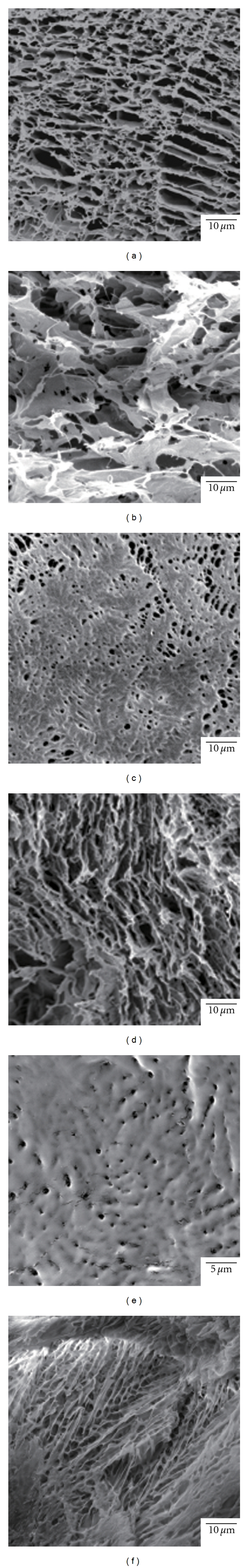
Electron microscopic images of the surface and interior of as-prepared FDPLLA particles. (a) *C*_0_ = 2.0 wt%, surface, (b) *C*_0_ = 2.0 wt%, interior, (c) *C*_0_ = 5.0 wt%, surface, (d) *C*_0_ = 5.0 wt%, interior, (e) *C*_0_ = 8.0 wt%, surface, and (f) *C*_0_ = 8.0 wt%, interior.

The yielded FDPLLA particles were revealed to be very porous.  Table 1 shows specific surface area *σ* and void fraction *r*_*v*_ (volume fraction of void) of FDPLLA particles. The void content was evaluated from averaged values of apparent radius and mass of the particle. It is noted from Table 1 that for amorphous FDPLLA, *C*_0_ + *r*_*v*_ approximately equals 100%. This means that the shape of the as-freeze-dried sphere is originated from the solution droplet: the frozen droplets undergo no shrinkage upon freeze drying.  Figure 2 shows scanning electron micrographs for FDPLLA particles. We see that the porosity is greater for lower *C*_0_. The thickness of the thin membranes that form fine tissue in the interior was estimated to be 100–200 nm from electron microscopy, which coincides with the results of specific surface area: thickness of a hypothetical flat film *d*_*f*_ that has the same specific surface area was estimated as *d*_*f*_ = 2/(*ρσ*), where *ρ* is the density of amorphous PLLA, and it ranged from 120 to 191 nm (see Table 1).

Figure 2 also shows that the surface of the sphere is less porous than that in the interior. Fine tissue that is typically seen in the interior appears to have melted on the surface, which results in reduction of surface porosity. This is typically observed in Figures 2(c) and 2(e). It appears that a less porous skin is formed on the surface. By analyzing the SEM images, we estimated surface porosity as void fraction of the surface, *A*_void_/*A*_*t*_, where *A*_void_ is the area of the voids (pores) on the surface, and *A*_*t*_ is the total area of the surface. The results are presented in Table 2. The surface porosity decreases with increasing *C*_0_, and it is extremely low for *C*_0_ = 8.0 wt%. The formation of the surface skin layer may have occurred during freezing. When a droplet of the solution is quenched in liquid N_2_, solid-liquid microphase separation would occur first, that is, crystallized solvent (solid phase) and unfrozen solution (liquid phase) may be formed. As the freezing process proceeds, the liquid phase becomes concentrated and finally it vitrifies when most of the solvent has moved to the frozen phase. Although the whole frozen process occurs within a few seconds in liquid N_2_, the intermediate liquid phase may have enough time to relax by reducing its interfacial area, which leads to surface melting. This process may occur preferentially on the surface where free space for the relaxation is available.

**Figure 3:**
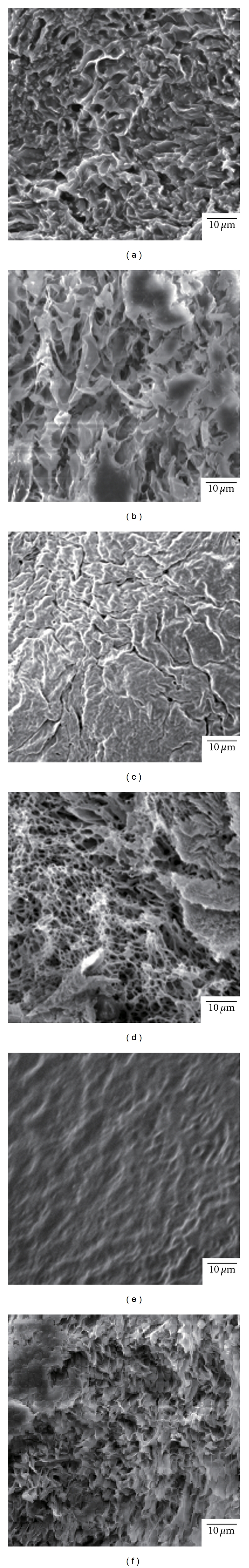
Electron microscopic images of the surface and interior of BSA-loaded FDPLLA particles. (a) *C*_0_ = 2.0 wt%, surface, (b) *C*_0_ = 2.0 wt%, interior, (c) *C*_0_ = 5.0 wt%, surface, (d) *C*_0_ = 5.0 wt%, interior, (e) *C*_0_ = 8.0 wt%, surface, and (f) *C*_0_ = 8.0 wt%, interior.

The mass fraction of BSA loaded in the FDPLLA is presented in Table 3, which is defined as *f*_BSA_ = *m*_BSA  _/*m*_*t*_, where *m*_BSA  _ is the mass of BSA adsorbed in FDPLLA, and *m*_*t*_ is the total mass of BSA-loaded FDPLLA. The obtained values of *f*_BSA_ are extremely large compared with the PLLA scaffold prepared by the phase separation method [22]. This feature is originated from the high porosity of the present FDPLLA, which seems to be suitable for drug delivery applications.  Figure 3 shows morphology of the BSA-loaded FDPLLAs. The interior of the particle exhibits a similar feature of porous morphology to that of the unloaded FDPLLA. The loaded BSA in the interior of the particle exists as agglomerates, which is typically seen in Figure 3(b). The high porosity for the BSA-loaded FDPLLA is reasonably understood from their void fraction *r*_*v*,  loaded_ presented in Table 3: it is rather high even after the BSA adsorption, indicating that the BSA-loaded FDPLLA is still very porous. Here, *r*_*v*,  loaded_ was estimated from *f*_BSA_ as

(1) $$\begin{array}{cc} r_{v,\text{loaded}} & =1-\;\left(1-r_{v}\right)\;\left(1+\frac{f_{\text{BSA}}}{1-f_{\text{BSA}}}\frac{\rho_{\text{PLLA}}}{\rho_{\text{BSA}}}\right) \end{array},$$

where *r*_*v*_ is the void fraction before the BSA loading, and *ρ*_PLLA_ and *ρ*_BSA_ (1.07 g cm^−3^) are the densities of PLLA and BSA, respectively. On the other hand, the surface of the particle seems to be covered with deposited BSA, and the voids on the surface is hidden for *C*_0_ = 5.0 and 8.0 wt%. From electron micrographs, the deposited BSA layer on the surface for *C*_0_ = 5.0 and 8.0 wt% was roughly estimated to be as thin as 0.5–1 *μ*m, which does not alter the apparent size of the particle significantly. As for *C*_0_ = 2.0 wt%, the surface is still ragged, which may be originated from the very porous surface shown in Figure 2(a).

**Figure 4:**
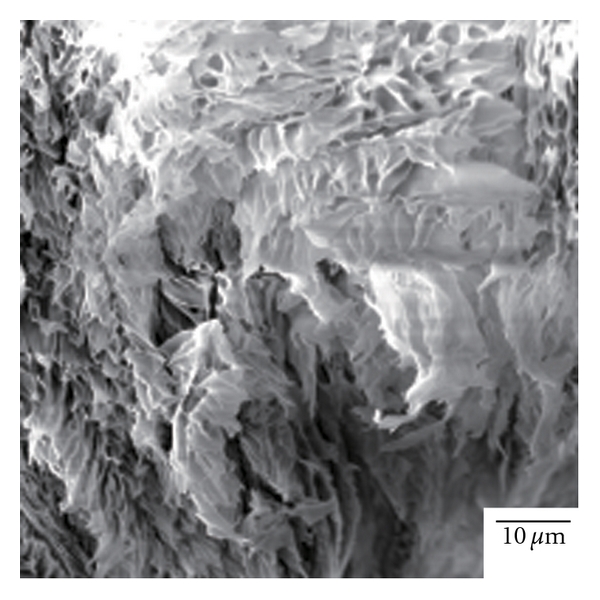
Electron microscopic image of the interior of crystallized FDPLLA particle with *C*_0_ = 8.0 wt%.

We found that the apparent size of FDPLLA particles is reduced upon isothermal crystallization at 115°C for 15 min. This tendency was remarkable for *C*_0_ = 2.0 wt%. Additionally, the spherical shape was deformed into indefinite shape. The apparent averaged radius is presented in Table 1 in the parentheses. This is just a rough estimation, because the particles were not spherical.  Table 1 also shows *r*_*v*_ values estimated from this averaged radius.  Figure 4 shows a typical morphology of the interior of crystallized FDPLLA (*C*_0_ = 8.0 wt%). Aggregation of curved fine layers is observed; each layer is probably made of stacked crystalline lamellae of PLLA. The crystallinity of these samples was revealed to be in the range 0.14–0.16. The space (void) between them was found to be reduced upon crystallization, which is consistent with the reduction of the apparent size of the particle (see Table 1). As for the BSA loading, *f*_BSA_ is smaller than that for the corresponding amorphous FDPLLA as shown in Table 3. It is most likely that this is due to the reduction of void fraction upon crystallization.

### 3.2. Release Kinetics

**Figure 5:**
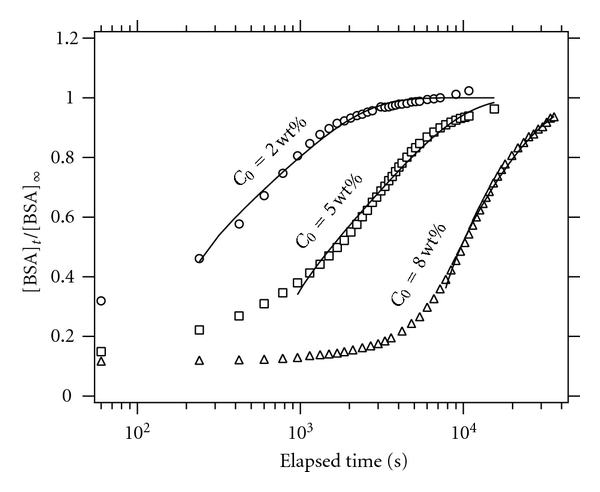
BSA release [BSA]_*t*_/[BSA]_*∞*_ at 37°C plotted against elapsed time. The solid curves indicate the results of the nonlinear least squares fitting analysis.

As the crystallized FDPLLA particles have indefinite shape which seems to prevent simple kinetic analysis, we here report the release data only from the amorphous FDPLLA. The amount of BSA released into water from the FDPLLA was evaluated by UV absorbance at 279 nm.  Figure 5 shows release of BSA with respect to elapsed time as presented by [BSA]_*t*_/[BSA]_*∞*_, where [BSA]_*t*_ is the BSA concentration in water at time *t*, and [BSA]_*∞*_ is the final value of the BSA concentration that was estimated from the total mass of BSA loaded in the FDPLLA. It is clearly seen that the release rate increases as *C*_0_ decreases. For the three samples, a major portion of BSA release occurs within at least several hours, which suggests that the release occurs mainly by the diffusion mechanism prior to the degradation of PLLA: we confirmed that significant hydrolysis of PLLA in water under the present condition takes more than several days.

We infer that the *C*_0_ dependence of release rate may be due to the difference in surface porosity. Additionally, it is seen that a burst of release occurs in the early period, which is probably due to rapid solvation of BSA deposited on the surface of the particles as observed in Figures 3(a), 3(c), and 3(e). We speculate that the release of BSA from the interior of the particle occurs in two subsequent processes: permeation of water into the FDPLLA sphere, and the diffusion of BSA out of the particle. Both of these processes are affected significantly by the surface porosity. The values of *A*_void_/*A*_*t*_ in Table 2 indicate that the surface porosity decreases remarkably with increasing *C*_0_. The interior porosity of the particle also decreases with increasing *C*_0_ as shown in Table 1, but this dependence is weaker than that of the surface porosity. Thus, it is likely that the release rate due to the diffusion process from the interior is largely governed by the surface porosity.

The profiles in Figure 5 suggest that the release takes place in two steps: dissolution of the BSA deposited on or near the surface occurs first, which is responsible for the initial burst, and then, BSA leaches out from the interior of the particle. The first step occurs within a few minutes, as evidenced by nonzero values of [BSA]_*t*_/[BSA]_*∞*_, at 60 s in Figure 5 (0.1–0.3). The release of the second step commences after certain induction period, which is needed for water to permeate into the interior of the particle, and this depends strongly on the surface porosity as discussed above. The release rate due to the second step is governed by the diffusion process of BSA in the particle and through the surface skin with less porosity.

To analyze the time-evolution profiles in Figure 5, we assume a model of Fickian diffusion from a nonswellable sphere [23]. In this case, an analytical expression for [BSA]_*t*_/[BSA]_*∞*_ is given by

(2) $$\frac{{\left[\text{BSA}\right]}_{t}}{{\left[\text{BSA}\right]}_{\infty }}=1-\frac{6}{\pi^{2}}\overset{\infty }{\underset{n=1}{\sum }}\frac{1}{n^{2}}\exp\left[-\frac{Dn^{2}\pi^{2}t}{a^{2}}\right]\;,$$

where *D* is the diffusion coefficient of BSA travelling in the FDPLLA particle, and *a* is the radius of the FDPLLA sphere. We further takes into account the initial burst as a baseline fraction parameter *b*, and the induction period for the second step as *t*_*i*_, thus, the time-dependent release is expressed by

(3) $$\begin{array}{cc} & \frac{{\left[\text{BSA}\right]}_{t}}{{\left[\text{BSA}\right]}_{\infty }}\\ & =\left(1-b\right)\;\left\{1-\frac{6}{\pi^{2}}\overset{\infty }{\underset{n=1}{\sum }}\frac{1}{n^{2}}\exp\left[-\frac{Dn^{2}\pi^{2}\left(t-t_{i}\right)}{a^{2}}\right]\;\right\}+b. \end{array}$$

The experimental profiles were fitted with equation (3) by nonlinear least squares fitting, *D* and *t*_*i*_ being treated as variable parameters, and the obtained best fit values are listed in Table 4. In the analysis, the series in equation (3) was cut off at *n* = 400; we confirmed that this does not affect the results significantly. Equation (3) assumes that both the distribution and diffusion rate of BSA are spatially uniform in the spherical particle, thus the evaluated *D* is regarded as an averaged diffusion coefficient over the interior of the sphere including the surface skin layer.

The diffusion coefficient *D* for *C*_0_ = 2.0 wt% is more than an order of magnitude greater than that for *C*_0_ = 8.0 wt%. This can be understood by the large difference in the surface porosity between the two samples (Table 2). Thus, it is reasonable to consider that the release rate due to the diffusion process is largely affected by the porosity of the surface skin layer. The baseline parameter *b* decreases with increasing *C*_0_, which implies that the release due to the initial burst is larger for lower *C*_0_. This suggests that the initial burst is originated not only from the BSA portion deposited on the surface but also from that deposited near the surface. The amount of the latter portion may be greater for higher surface porosity. The induction time *t*_*i*_ increases with increasing *C*_0_. We infer that during the induction period, water goes into the particle, and this process is considered to be affected strongly by the surface porosity.

## 4. Conclusions

In this study, we have prepared BSA-loaded FDPLLA particles and have investigated their morphology and release kinetics of BSA into water. We have found that the porosity and specific surface area of the present FDPLLA are rather high, which leads to prominent ability of protein loading. The surface of FDPLLA particle is less porous than the interior, which is suggested to affect significantly the release rate of BSA. The concentration *C*_0_ of the original solution, from which FDPLLA is prepared, seems to be a key parameter that controls both BSA loading and release rate: as *C*_0_ decreases, the loading of BSA increases, while the release rate becomes greater.

The present results suggest that FDPLLA is a promising material as a carrier for controlled drug release. On the other hand, it may not be suitable for tissue engineering applications, because the size of the pore is too small for cell seeding, growth, and tissue formation. The release rate observed in this study may be too fast for a practical use in controlled releasing system. This problem could be overcome by arranging the surface of the FDPLLA particle by either chemical or physical treatment. In addition, the size of the particle could be arranged so as to be suitable for various medical administrations (ingestion or injection) by development of spray method on freezing with controlled viscosity of the solution. We are currently exploring these techniques.

## Figures and Tables

**Table 1:** Averaged radius *a*, specific surface area *σ*, and void content *r*_*v*_ of FDPLLA particle.

*C*_0_ (wt%)		*a* (mm)	*σ* (m^2^ g^−1^)	*d*_*f*_ (nm)^a^	*r*_*v*_ (vol%)
2.0	Amorphous	1.47 ± 0.03	13.4	120	98.1
2.0	Crystallized	(0.8)			87.5
5.0	Amorphous	1.58 ± 0.07	11.9	135	94.4
5.0	Crystallized	(1.1)			85.0
8.0	Amorphous	1.49 ± 0.11	8.4	191	92.3
8.0	Crystallized	(1.1)			80.2

^a^Hypothetical membrane thickness estimated as *d*_*f*_ = 2/(*ρσ*).

**Table 2:** Void fraction on the surface, *A*_void_/*A*_*t*_ of FDPLLA particle.

*C*_0_ (wt%)		*A*_ void_/*A*_*t*_(%)
2.0	Amorphous	67.3
5.0	Amorphous	10.7
8.0	Amorphous	3.0

**Table 3:** Mass fraction of BSA *f*_BSA_ and void fraction *r*_*v*,loaded_  of BSA-loaded FDPLLA.

*C*_0_ (wt%)		*f*_BSA_ (wt%)	*r*_*v* ,loaded_ (vol%)
2.0	Amorphous	79.1	89.7
2.0	Crystallized	53.0	71.1
5.0	Amorphous	63.4	83.1
5.0	Crystallized	43.8	71.4
8.0	Amorphous	51.3	82.8
8.0	Crystallized	34.5	68.0

**Table 4:** Parameters obtained from the analysis of [BSA]_*t*_/[BSA]_*∞*_ versus time.

*C*_0_ (wt%)	*b*	*t*_*i*_(s)	*D* (m^2^ s^−1^)
2.0	0.32	2.0 × 10^2^	2.1 × 10^−10^
5.0	0.15	7.5 × 10^2^	6.0 × 10^−11^
8.0	0.13	6.7 × 10^3^	1.7 × 10^−11^
